# Identification of a Potential Common Ancestor for Mammalian Cross-Presenting Dendritic Cells in Teleost Respiratory Surfaces

**DOI:** 10.3389/fimmu.2018.00059

**Published:** 2018-01-25

**Authors:** Irene Soleto, Uwe Fischer, Carolina Tafalla, Aitor G. Granja

**Affiliations:** ^1^Centro de Investigación en Sanidad Animal (CISA), INIA, Valdeolmos, Spain; ^2^Bundesforschungsinstitut für Tiergesundheit, Friedrich-Loeffler-Institut (FLI), Insel Riems, Germany

**Keywords:** dendritic cells, rainbow trout, gills, respiratory surfaces, cross-presentation, CD8, major histocompatibility complex II

## Abstract

Dendritic cells (DCs) are highly specialized antigen-presenting cells that bridge innate and adaptive immune responses in vertebrates, being key modulators in the initiation of specific responses. Although teleost fish present the main elements of a fully developed adaptive immune system, not many studies have focused on identifying specific DC subsets in teleost species. Previous work from our group identified in rainbow trout (*Oncorhynchus mykiss*) skin a DC subpopulation co-expressing CD8α and major histocompatibility complex II β on the cell surface. Interestingly, these CD8^+^ DCs expressed common unique markers of mammalian cross-presenting DCs, a DC subset with an important role in antigen presentation and activation of CD8^+^ T cytotoxic lymphocytes. In this study, we have identified a similar DC subset in rainbow trout gills that also transcribes molecules uniquely expressed on diverse mammalian cross-presenting DC populations such as CD8, CD103, CD141, Batf3, IFN regulatory protein 8, and toll-like receptor 3. Hence, we have undertaken a broad phenotypic and functional characterization of this new DC subset that includes the confirmation of novel capacities for DCs in teleost, such an IgM-binding capacity and responsiveness to CD40 ligand. Furthermore, our results show that in gills, this DC subset shows some different phenotypic and functional characteristics when compared with their homologs in the skin, suggesting an adaptation of the cells to different mucosal tissues or different maturation status depending on their location. Our findings contribute to increase our knowledge on fish cross-presenting DCs, an important cell population to take into account for the future design of mucosal vaccination strategies.

## Introduction

Dendritic cells (DCs) are professional antigen-presenting cells (APCs) that play a key role orchestrating adaptive immune responses. DCs are found in most tissues but are particularly abundant in mucosal interfaces to induce specific immunity against invading pathogens and maintain tolerance to self- or innocuous antigens (1). DCs express a wide range of pattern recognition receptors including toll-like receptors (TLRs) that allow them to sense foreign antigens. Upon sensing, they have the capacity to internalize antigens, process them and present them in the context of major histocompatibility complex (MHC) I or MHC II to T cells (2). Unlike other phagocytes such as macrophages or neutrophils, DCs are not involved in pathogen clearance. Thus, phagosomal degradation and acidification are much lower in DCs than in other phagocytes, and consequently the antigen is best preserved and a wider range of peptides are presented in MHC I and MHC II molecules, being this the reason for the increased capacity of DCs to prime and stimulate T cells (3).

Although teleost fish constitute the first animal group in which all the main elements of a complete adaptive immune system are present, only a few studies have addressed the characterization of DCs in fish. In salmonids, several studies suggested the presence of DCs such as those describing cells containing Birbeck granules (4), cells with DC-like morphology (5) or cells expressing transcripts of DC markers such as CD208/LAMP3 (6). Later on, a mammalian protocol was adapted in rainbow trout (*Oncorhynchus mykiss*) to obtain hematopoietic cultures enriched in cells that shared many DC characteristic features and showed a greater capacity than B cells or macrophages to stimulate T cell proliferation (7). In zebrafish (*Danio rerio*), a leukocyte subpopulation enriched from lymphoid tissues by affinity to the lectin peanut agglutinin was also shown to share morphological and functional features of mammalian DCs (8). Also in zebrafish, CD209/DC-SIGN expression defined a cell type that co-expressed MHC II, CD80/86, and CD83 and was able to initiate antigen-specific CD4^+^ T cell activation (9).

All these studies confirmed the presence of DCs in teleost, but it was not until 2015 that a specific DC subset was identified in teleost fish (10). This specific subpopulation identified in rainbow trout skin consisted in large granular cells defined by high surface expression levels of MHC II and surface expression of CD8α. These cells contained negligible amounts of T cell transcripts (CD3, TCRα, and TCRβ); showed high transcription levels of characteristic DC markers such as DC-SIGN or LAMP3; were phagocytic for both polystyrene beads and apoptotic cells; were responsive to TLR ligands and to *in vivo* stimulation; and presented a semi-mature profile with high levels of CCR7 surface expression and intermediate levels of CD83 and co-stimulatory molecules (10). Interestingly, these CD8^+^ DCs found in rainbow trout skin expressed common unique markers of mammalian cross-presenting DCs, strongly supporting the hypothesis of them being a common ancestor for vertebrate cross-presenting DCs.

Generally, intracellular antigens (generated by virus infection or tumor development) are presented to cytotoxic CD8^+^ T cells in the context of MHC class I molecules, whereas extracellular antigens are taken up by APCs that present them in an MHC II context to helper CD4^+^ T cells. In addition, specific subsets of DCs, even when not infected, are able to acquire extracellular antigens, process them, and present them in the context of MHC I through a process designated as cross-presentation (11). These cross-presenting DCs are more efficient than other DC subsets in triggering effective T cytotoxic responses against intracellular pathogens and tumors (12). In mice, cross-presenting DCs include resident CD8^+^ DCs found in spleen, lymph nodes (LNs), and thymus (13) and migratory CD103^+^ DCs derived from tissues such as skin, lung, and intestine (14). In humans, a CD141^hi^ DC subset identified in blood (15) and tissues such as dermis, and liver and lung (16) was found to have a superior capacity to cross-present than other human DC populations. Interestingly, all these cross-presenting DC populations from mice and human share common exclusive features, which are not found in other DC subsets, thus suggesting the existence of a common ancestor. For instance, all cross-presenting populations use the CLEC9A lectin to recognize necrotic cells (16–18) and express the chemokine receptor XCR1 (19) and TLR3 to respond to viral stimuli (16, 20, 21). Furthermore, the functionality of all these cross-presenting populations is regulated by the fms-like tyrosine kinase 3 ligand, IFN regulatory protein 8 (IRF8) (22, 23), and Batf3 (24, 25). Remarkably, the identification of a subpopulation of DCs in teleost skin expressing CD8, CD103, CD141, TLR3, IRF8, and Batf3 strongly pointed to these cells as a potential common ancestor for mammalian cross-presenting DCs (10).

Because cross-presenting DCs had also been identified in human lungs, in the current work, we explored whether a CD8^+^ DC subset similar to that found in rainbow trout skin could also be identified in teleost gills, an equivalent respiratory organ. Lungs and gills are specialized respiratory surfaces that have evolved in different organisms in a quite specific manner depending on whether oxygen had to be taken up from the air or the water, their behavioral activities or their phylogenetic level of development (26). Despite these anatomical differences, all respiratory surfaces contain a specialized associated immune system that constitutes a first line of defense against air- or waterborne infectious agents. In this context, our study reports the identification of a specific DC subset for the first time in teleost gills. Similarly to their skin counterpart, these gill CD8^+^ DCs were capable of undertaking DC-specific activities and also expressed specific markers of different mammalian cross-presenting DC subsets. In addition, our studies have revealed novel capacities for DCs in teleost, such as an IgM-binding capacity and responsiveness to CD40 ligand (CD40L).

## Materials and Methods

### Experimental Fish

Female rainbow trout (*O. mykiss*) of ~50 g were obtained from Piscifactoría Cienfuentes (Guadalajara, Spain) and maintained at the animal facilities of the Centro de Investigación en Sanidad Animal (CISA-INIA) in an aerated recirculating water system at 16°C, with 12:12 h light/dark photoperiod. Fish were fed twice a day with a commercial diet (Skretting). Before any experimental procedure, fish were acclimatized to laboratory conditions for at least 2 weeks. All of the experiments described comply with the Guidelines of the European Union Council (2010/63/EU) for the use of laboratory animals and have been approved by the Instituto Nacional de Investigación Agraria y Alimentaria (INIA) Ethics Committee (ORCEEA 2016-021).

### Tissue Sampling

Rainbow trout were killed by benzocaine (Sigma) overdose and gills and spleen collected. Single cell suspensions were obtained using 100 µm nylon cell strainers (BD Biosciences) and Leibovitz medium (L-15, Invitrogen) supplemented with 100 IU/ml penicillin and 100 µg/ml streptomycin (P/S, Life Technologies), 10 U/ml heparin (Sigma), and 5% fetal calf serum (FCS, Life Technologies). Cell suspensions were placed onto 30/51% discontinuous Percoll (GE Healthcare) density gradients and centrifuged at 500 × *g* for 30 min at 4°C. The interface cells were collected and washed twice in L-15 containing 5% FCS.

### Flow Cytometry

For the identification of DC populations, leukocytes were incubated for 30 min with anti-trout CD8α (mAb rat IgG_2_; 7 µg/ml) (27) and anti-trout MHC II [mAb mouse IgG_1_ coupled to allophycocyanin; 2 µg/ml] (10) antibodies in L-15 media supplemented with 5% FCS. Cells were then washed twice with culture media and stained for 20 min with a secondary Ab for anti-CD8α [R-phycoerythrin F(ab′)_2_ fragment of goat anti-rat IgG (H + L) (Life Technologies)] in L-15 media supplemented with 5% FCS. After incubation, cells were washed two times with L-15 with 5% FCS and analyzed on a FACSCalibur flow cytometer (BD Biosciences) equipped with CellQuest Pro software.

To determine the levels of expression of surface CCR7 in CD8^+^ DC populations, the anti-trout CD8α and anti-trout MHC II (allophycocyanin-labeled) antibodies were combined with a specific anti-CCR7 polyclonal antibody (pAb rabbit IgG; 2 µg/ml) (28). After 30 min, the cells were washed twice with culture media and stained 20 min with secondary antibodies that included an R-phycoerythrin F(ab′)_2_ fragment of goat anti-rat IgG (H + L) and an Alexa Fluor^®^ 488 F(ab′)_2_ fragment of goat anti-rabbit IgG (H + L) (Life Technologies). After incubation, cells were washed two times with L-15 with 5% FCS and analyzed on a FACSCalibur flow cytometer.

In all cases, isotype controls for mouse mAbs, rat mAb, and rabbit pAb (BD Biosciences) were tested in parallel to discard unspecific binding of the Abs, and cells were stained with propidium iodide (PI; 0.5 µg/ml) to check cell viability. Flow cytometry analysis was performed with FlowJo 10 (TreeStar).

### Confocal Microscopy

Rainbow trout gills from anesthetized and exsanguinated rainbow trout were embedded in PolyFreeze cryostat mounting medium (Sigma), immediately frozen in liquid nitrogen, and stored at −80^○^C until used. Cryostat sections with a thickness of 14 µm were prepared using a Leica CM3050 microtome and placed on SuperFrost glass slides (Menzel-Gläser). Dry sections were fixed in acetone at −20°C for 20 min, air dried, encircled with a hydrophobic compound (ImmunoPen; Calbiochem), incubated for 1 h at room temperature with a blocking solution (TBS buffer pH 7.5, containing 0.01% BSA, 0.5% saponin, 0.02% Tween-20, and 5% goat serum), and stained with Abs against trout MHC II β-chain (allophycocyanin-labeled) and trout CD8α. Samples were washed and incubated with Alexa Fluor 488 F(ab′)_2_ fragment of goat anti-rat igG (H + L) (Life Technologies). Samples were counterstained with 1 µg/ml DAPI (Sigma). Laser scanning confocal microscopy three-dimensional image stacks (12 µm total thickness) were acquired with an inverted Zeiss Axiovert LSM 880 microscope. Images were analyzed with Zen 2.0 (Carl Zeiss) and Fiji (NIH) software packages.

### Transcriptional Analysis of FACS Isolated Populations

Gill CD8^+^ DC populations were isolated by flow cytometry in a BD FACSAria III cell sorter (BD Biosciences) after staining gill leukocytes with anti-trout CD8α and anti-trout MHC II antibodies as described earlier and using their FSC/SSC and fluorescence characteristics. Splenic IgM^+^ B cells and CD8^+^ cytotoxic T cells (lymphoid CD8^+^MHC II^−^ cells) were also isolated by flow cytometry in a BD FACSAria III cell sorter to be used in some specific experiments. For this, spleen leukocytes were incubated with anti-trout IgM mAb (1.14, mouse IgG1) coupled to APC or with a combination of anti-trout CD8α and anti-trout MHC II antibodies as previously described. RTS11, a rainbow trout monocyte/macrophage cell line established from spleen (29) was also included in some transcriptional analysis for comparative purposes.

Total cellular RNA was isolated from cell populations using the Power SYBR Green Cells-to-Ct Kit (Invitrogen) following the manufacturer’s instructions. RNA was treated with DNase during the process to remove genomic DNA that might interfere with the PCR reactions. Reverse transcription was also performed using the Power SYBR Green Cells-to-Ct Kit (Invitrogen) following the manufacturer’s instructions. To evaluate the levels of transcription of the different genes, real-time PCR was performed with a LightCycler^®^ 96 System instrument (Roche) using SYBR Green PCR core Reagents (Applied Biosystems) and specific primers (Table S1 in Supplementary Material). Each sample was measured in duplicate under the following conditions: 10 min at 95°C, followed by 40 amplification cycles (15 s at 95°C and 1 min at 60°C). A melting curve for each primer set was obtained by reading fluorescence every degree between 60 and 95°C to ensure only a single product had been amplified. The expression of individual genes was normalized to the relative expression of trout housekeeping gene elongation factor 1α (EF-1α), and the expression levels were calculated using the 2^−ΔCt^ method, where ΔCt is determined by subtracting the EF-1α value from the target Ct. No template negative controls and minus reverse transcriptase controls were included in all the experiments.

### Phagocytic Activity

To analyze the phagocytic capacity of gill CD8^+^ DCs, gill leukocytes were seeded in 24-well plates (Nunc) at a cell density of 1 × 10^6^ cells/well and incubated for 16 h at 20°C with fluorescent beads (FluoSpheres^®^ Microspheres, 1.0 µm, Crimson Red Fluorescent 625/645, 2% solids; Life Technologies) at a cell:bead ratio of 1:10 or without beads in the case of negative controls. After the incubation period, cells were harvested by gently pipetting, and non-ingested beads were removed by centrifugation (100 × *g* for 10 min at 4°C) over a cushion of 3% (weight/volume) BSA (Fraction V; Fisher Scientific) in PBS supplemented with 4.5% (weight/volume) d-glucose (Sigma). Cells were resuspended in L-15 with 5% FCS, labeled with the flow cytometry antibodies and analyzed on a FACSCalibur flow cytometer or under the confocal microscope. In some experiments, cytochalasin B (0.05 µg/ml) was added to the cells immediately before the addition of the beads to verify active phagocytosis.

### Mixed Leukocyte Reaction (MLR)

Gill CD8^+^ DCs were isolated by cell sorting as described earlier and cultured for 12 h in L-15 medium supplemented with 20% FCS in the presence of the T cell-dependent antigen TNP hapten conjugated to the keyhole limpet hemocyanin (TNP-KLH) (Biosearch Technologies). Because no antibodies are available against extracellular pan-T cell markers in rainbow trout, we used T cell-enriched cultures as responder cells. These T cell-enriched cultures were obtained from isogeneic splenocytes by depleting all IgD^+^, IgM^+^, and MHC II^+^ cells through cell sorting. The resulting negative population, representing approximately 10% of splenocytes, was then labeled with Cell Trace™ CFSE Cell Proliferation Kit (ThermoFisher Scientific). The enrichment in T cells was verified by stimulation with Concanavalin A (ConA, 4 µg/ml; Sigma), a typical T cell mitogen. To carry out the MLR, stimulated DCs were cocultured with isogeneic T cell-enriched splenocytes at a ratio of 1:30 (DCs:splenocytes). After 5 days of incubation at 20°C, cocultured samples were stained with 7-AAD (BD Biosciences) at 2.5 µg/ml to check cell viability and analyzed by flow cytometry to measure cell proliferation of the enriched T cell population through the degree of dilution of CFSE.

### IgM-Binding Capacity

We assessed the IgM-binding capacity of gill CD8^+^ DCs. For this, rainbow trout IgM was purified from serum by affinity chromatography, using Econo-Column Chromatography columns (BIO-RAD) and CNBr-activated Sepharose 4b (GE Healthcare), coated with anti-trout IgM mAb (1.14), according to the manufacturer’s instructions. Thereafter, gill leukocytes were incubated with 0.1 µg/ml purified IgM for 1 h at 20°C. Subsequently, cells were washed twice with L-15 with 5% FCS and then stained with anti-trout CD8α and anti-trout MHC II β-chain as described earlier, in combination with an anti-trout IgM [mAb 1.14 mouse IgG_1_ coupled to fluorescein (FITC); 1 µg/ml] (30). Cells were washed twice, resuspended in L-15 with 5% FCS, and analyzed on a FACSCalibur flow cytometer.

### CD40L Stimulation

The nucleotide sequence corresponding to the extracellular domain of the rainbow trout CD40L (GenBank Accession number EF160131) together with an N-terminal 6× histidine tag was synthetized and subcloned into the E3 expression vector (Abyntek). The recombinant plasmid was transformed into BL21 cells, and kanamycin-resistant single positive colonies for each clone were then incubated at 37°C in Luria–Bertani media. When the OD_600_ reached 0.6, 0.1 mM of isopropyl β-d-thiogalactoside (IPTG, Sigma Aldrich) was added to induce protein production. After 16 h, cells were harvested, lysed by sonication and dissolved using urea. Thereafter, recombinant CD40L was obtained through the use of Nickel columns (Sigma Aldrich). The protein-containing fractions were pooled, refolded, filtered through 0.22 µm, and resuspended in storage buffer (50 mM Tris–HCl, 150 mM NaCl, 10% glycerol, and 0.5 M l-arginine, pH 9.0). Protein concentrations were determined in a BCA protein assay (ThermoFisher Scientific), and the recombinant rainbow trout CD40L (85% purity) aliquoted and stored at −80°C until used. An irrelevant protein with a similar molecular weight also bearing an N-terminal His tag was produced in the same conditions and was used as a functional control (C-His).

To establish the effect of CD40L on gill CD8^+^ DCs, gill leukocytes were cultured with 5 µg/ml CD40L, with the same concentration of C-His or with media alone. This dose had been previously optimized in functional assays performed with B cells in our laboratory (data not shown). After 48 h of incubation at 20°C, the percentage of CD8^+^ DCs and their levels of expression of surface MHC II were analyzed by flow cytometry as described earlier.

### Statistical Analysis

Statistical analyses were performed using a two-tailed Student’s *t* test with Welch’s correction when the *F* test indicated that the variances of both groups differed significantly. The differences between the mean values were considered significant on different degrees, where * means *p* ≤ 0.05, ** means *p* ≤ 0.01, and *** means *p* ≤ 0.005.

## Results

### Identification of CD8^+^ DCs in the Gill of Rainbow Trout

**Figure 1:**
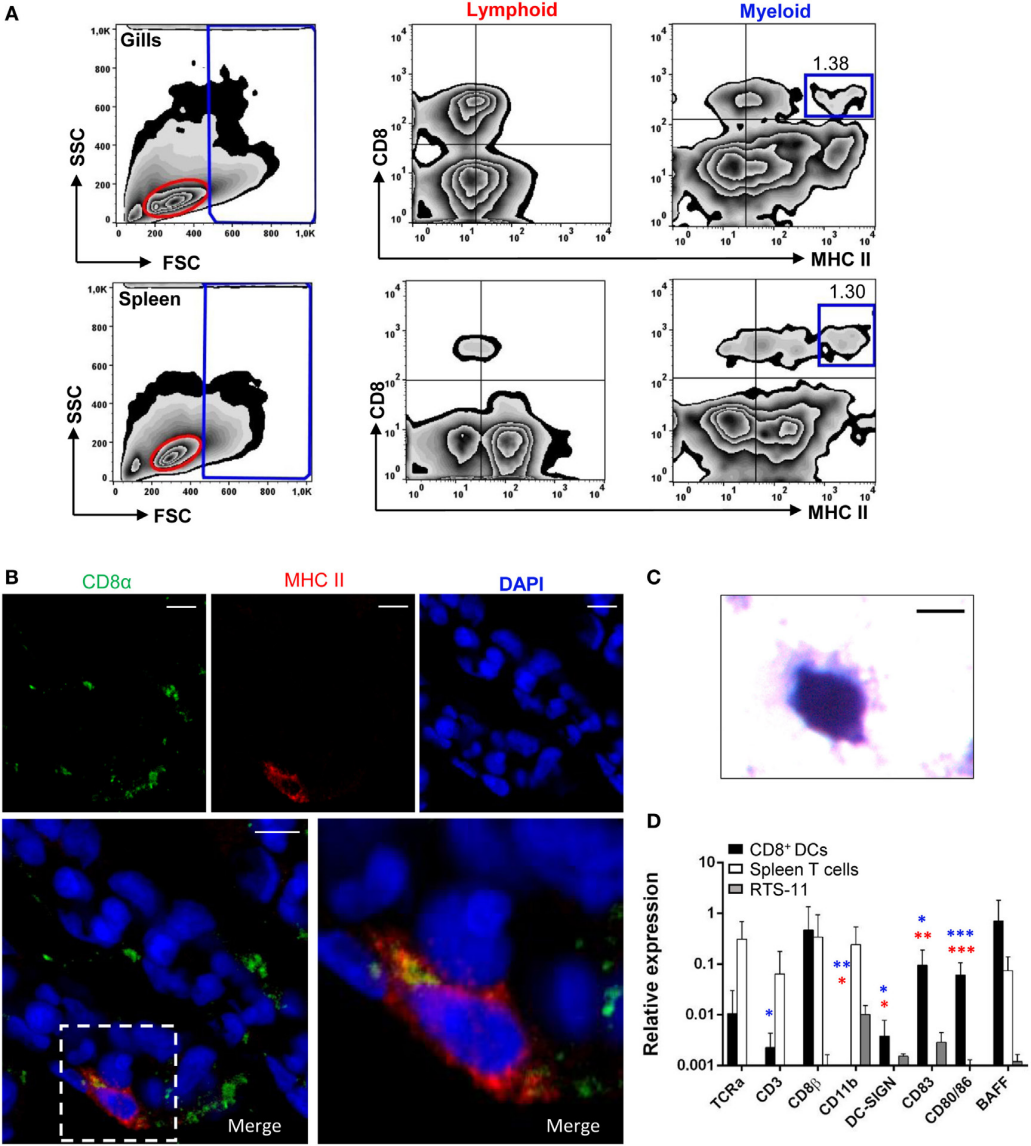
Identification and characterization of gill CD8α^+^ major histocompatibility complex (MHC) II^+^ cells. **(A)** Flow cytometry analysis of rainbow trout leukocytes isolated from gills (top panels) and spleen (bottom panels) stained with anti-CD8α and anti-MHC II mAbs. FSC/SSC profiles are shown (left) and gates for lymphoid (L) and myeloid (M) cells defined. Two-color CD8/MHC class II dot plots of lymphoid and myeloid gated cells are also shown. Percentage of myeloid CD8^+^MHC II^hi^ cells among the total number of cells in each gate is shown in the upper right corner. **(B)** Cryostat sections were prepared from rainbow trout gill, fixed and labeled with anti-CD8α (green) and anti-MHC class II (red) Abs, counterstained with DAPI (blue), and analyzed by fluorescence microscopy. A representative image is shown, together with a magnification of a CD8^+^ dendritic cell (DC) (right). **(C)** CD8α^+^MHC II^+^ cells from gills were isolated by cell sorting and then incubated onto poly-l-lysine-treated glass slides, fixed, mounted, and analyzed by light microscopy (scale bar, 5 µm). **(D)** Gill CD8^+^ DCs and splenic CD8^+^ T cells were isolated by flow cytometry and RNA obtained. RNA was also obtained from the RTS11 monocyte–macrophage cell line. These RNAs were used to study the levels of transcription of different marker genes by real-time PCR. Relative expression of the indicated genes to the endogenous control elongation factor 1α was calculated for each sample value. Mean values (+SD) from three independent experiments are shown. Asterisks in red indicate significant differences between values obtained in gill CD8^+^ DCs and splenic CD8^+^ T cells, whereas asterisks in blue denote significant differences between values obtained in gill CD8^+^ DCs and RTS11 cells (*p ≤ 0.05, **p ≤ 0.01, and ***p ≤ 0.005).

Because in mammals cross-presenting DCs are also found in the lungs, we examined whether a CD8^+^ DC subset equivalent to that previously reported in rainbow trout skin (10) was also found in gills. For this, gill leukocytes were analyzed by flow cytometry, using anti-trout MHC II and anti-trout CD8α mAbs, and compared with the profile observed in splenocytes. Cells were first analyzed according to their FSC/SSC profile (Figure 1A, left panels), dividing the cells into those that are included within the lymphoid gate (FSC^low^SSC^low^) representing lymphocytes, or within what we have cataloged as a myeloid gate (FSC^high^SSC^med/high^), representing larger and more complex cells, such as macrophages, neutrophils, and DCs (10, 31). Within the myeloid gate, a subset of cells expressing surface CD8 and high levels of surface MHC II cells were found in the gills, representing 1.29% of the cells in the myeloid gate (Figure 1A, top panels), in a similar trend to that seen on the spleen, where this subset was also found in a similar percentage (Figure 1A, bottom panels). Some cells expressing CD8^+^ and lower surface MHC II levels were also observed in both gills and spleen, but in this work we focused exclusively on those expressing high surface MHC II levels such as those previously identified in skin and designated as CD8^+^ DCs (10). CD8^+^MHC II^+^ cells were also found in the lymphoid population of gill leukocytes in high numbers, but this population was almost absent in the spleen. However, the characterization of this lymphoid CD8^+^MHC II^+^ subset was not the aim of this study, and it is something we will address in future investigations.

Confocal microscopy analysis of gill sections stained with fluorescently labeled antibodies against CD8 and MHC II revealed that these large CD8^+^ cells with high levels of MHC II were primarily located at the distal part of secondary gill filaments (Figure 1B; Figure S1 in Supplementary Material). To characterize the morphology of these cells, they were sorted by flow cytometry and analyzed by light microscopy (Figure 1C). Cells showed a round irregular morphology with small membrane projections, as previously described for mammalian DCs. To further characterize this CD8^+^ DC population identified in the gills, we analyzed the transcription levels of specific T cell and DC markers by real-time PCR comparing them to the transcription profiles obtained for CD8^+^ cytotoxic T cells from spleen (CD8^+^MHC II^−^ from the lymphoid gate) and the trout macrophage cell line RTS11. We observed that the gill CD8^+^ DCs expressed DC-SIGN (Figure 1D), confirming they constitute a DC subset. In addition, they also expressed very high levels of CD83, CD80/86, and BAFF when compared with CD8^+^ T cells or macrophages, also supporting their identification as a DC subpopulation. In concordance, these CD8^+^ DCs express very low levels of TCRα and CD3 compared with the levels obtained in spleen CD8^+^ T cells (approximately 100-fold lower). Interestingly, the transcriptional profile obtained also pointed out some differences between gill CD8^+^ DCs and the equivalent subset previously identified in trout skin. For example, gill CD8^+^ DCs did not express CD11b whereas skin CD8^+^ DCs did; and showed expression levels of CD8β comparable with those found on CD8^+^ T cells, indicating that this DC subset was CD8α/β, contrarily to the skin DC subset, which was reported as CD8α/α (10).

### Phagocytic Capacity of Teleost Gill CD8^+^ DCs

**Figure 2:**
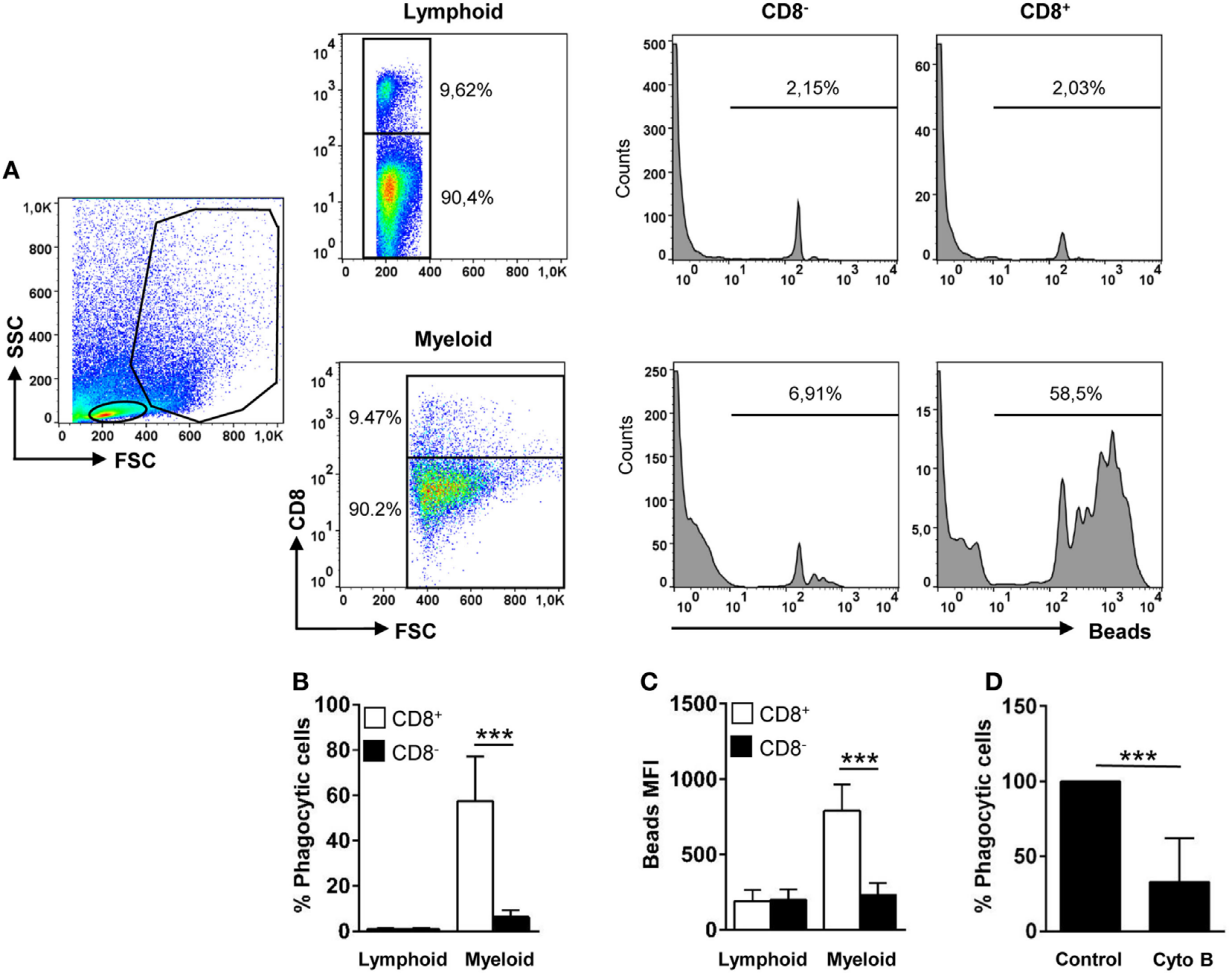
Phagocytic capacity of gills CD8^+^ dendritic cells (DCs). Leukocytes from gills were incubated with Crimson Red fluorescent polystyrene beads (1 µm diameter) at a ratio of 1:10 (cell/beads) for 16 h. Non-ingested beads were removed in a glucose gradient, and the cells were stained with anti-CD8α and analyzed by flow cytometry **(A)**. Lymphoid cells (upper panels) and myeloid cells (lower panels) were gated, and CD8^−^ and CD8^+^ cells were further selected to analyze and measure the fluorescence of internalized beads (histograms). The percentage of cells containing beads was determined using control samples without beads to establish the phagocytosis gate shown in the histograms. The average percentage of phagocytic CD8^−^ and CD8^+^ cells **(B)** and the mean fluorescence intensity (MFI) of the internalized beads **(C)** were used to determine the phagocytic capacity of each cell type. Data are representative of 11 individual fish from 5 different experiments. **(D)** Cytochalasin (0.05 µg/ml) was added to the cells immediately before the addition of the beads to demonstrate active phagocytosis. Results are shown as the percentage of phagocytic gill CD8^+^ DCs relative to that of untreated controls (five individual fish) (***p ≤ 0.005).

Trout gill CD8^+^ DCs displayed a high phagocytic capacity, in contrast to lymphoid CD8^+^ cells that were not phagocytic (Figures 2A,B). After 16 h of incubation with 1 µm Crimson red-labeled polystyrene beads, ~60% of the CD8^+^ DCs had internalized beads, whereas only ~8% of the CD8^−^ fraction within the myeloid gate had internalized beads (Figures 2A,B). In addition, the mean fluorescence intensity (MFI) of internalized beads within CD8^+^ DCs was significantly higher than that observed in CD8^−^ cells within the myeloid gate (Figure 2C), indicating that the average number of particles internalized per cell was much higher for CD8^+^ DCs than for other APCs present in the gills. The internalization of beads by trout gill CD8^+^ DCs was significantly inhibited by cytochalasin B demonstrating this is an active phagocytosis process that requires actin polymerization (Figure 2D). In addition, the fact that the beads detected in flow cytometry were located always inside the cells was also verified through confocal microscopy (Figure S2 in Supplementary Material). These results clearly point out that trout gill CD8^+^MHC II^+^ cells within the myeloid gate are, in fact, phagocytic CD8^+^ DCs.

### Teleost Gill CD8^+^ DCs Prime Proliferation of T Cells

**Figure 3:**
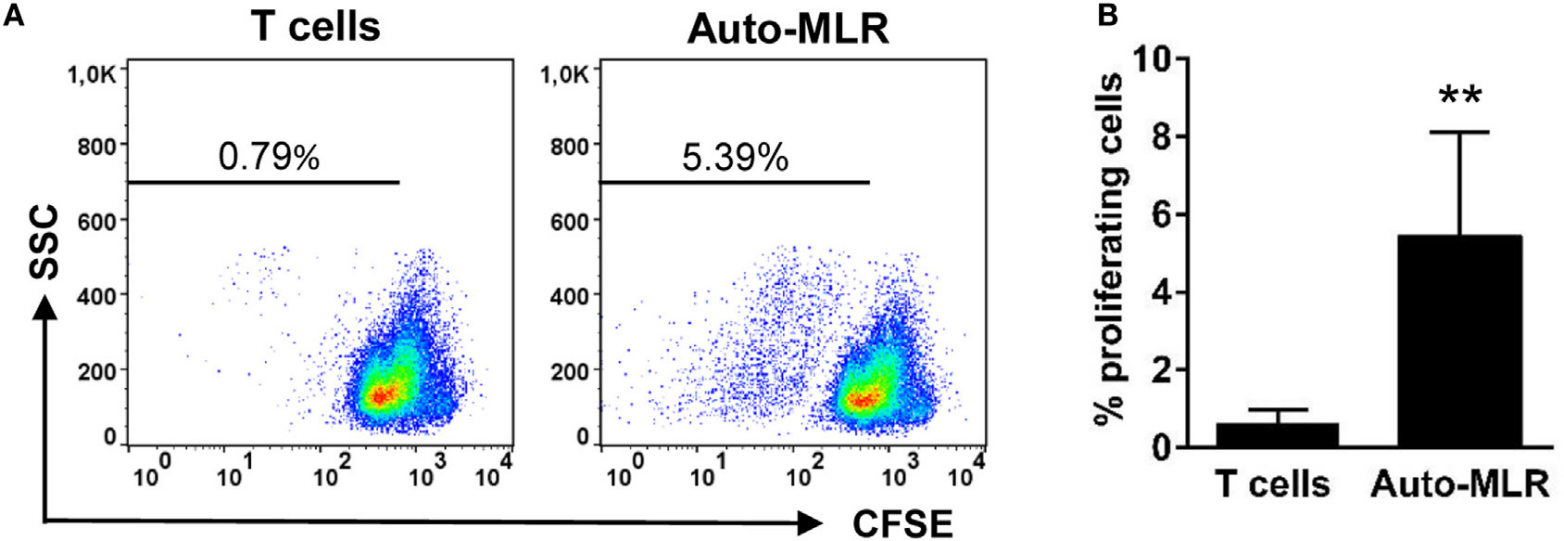
T cell-activating capacity of gill CD8^+^ dendritic cells (DCs). Trout gill CD8^+^ DCs were isolated by flow cytometry. Cells were then cultured for 12 h in L-15 medium supplemented with 20% fetal calf serum in presence of the T cell-dependent Ag TNP hapten conjugated to the keyhole limpet hemocyanin. Thereafter, DCs were cocultured with isogenic T cell-enriched fractions obtained from the spleen of these same animals [auto-mixed leukocyte reaction (MLR)], previously labeled with CFSE. Cocultures were set at a ratio of 1:30 (DC:T cells) and incubated for 5 days. After this time, the cultures were analyzed by flow cytometry to measure the level of CFSE dilution. **(A)** A representative example from eight individuals is shown, and the percentage of proliferating cells is indicated. **(B)** Average percentages of proliferating cells were calculated (*n* = 8, mean + SD) (**p ≤ 0.01).

One of the main defining features of DCs is their capacity to stimulate T cells (32). Thus, to demonstrate their T cell-activating potential, we performed an MLR. To carry this out, we FACS isolated gill CD8^+^MHC II^+^ cells and incubated them overnight in the presence of TNP-KLH (a T cell-dependent antigen). This CD8^+^ DC subpopulation was co-incubated with isogeneic (auto-MLR) splenocyte cultures enriched in T cells from which all APCs had been previously depleted. This enriched population strongly proliferated in the presence of ConA (Figure S1 in Supplementary Material), a specific T cell mitogen (33), thus confirming the T cell enrichment but was not able to proliferate in response to TNP-KLH alone (Figure S3 in Supplementary Material). However, when this T cell-enriched population was cocultured with gill CD8^+^ DCs, a significant T cell proliferation was detected by means of CFSE dilution, when compared with T cell-enriched splenocytes incubated in the absence of gill DCs (Figures 3A,B). These data demonstrate a capacity for the teleost gill CD8^+^ DC subpopulation to activate T lymphocytes.

### Gill CD8^+^ DCs Express High Levels of Surface CCR7

**Figure 4:**
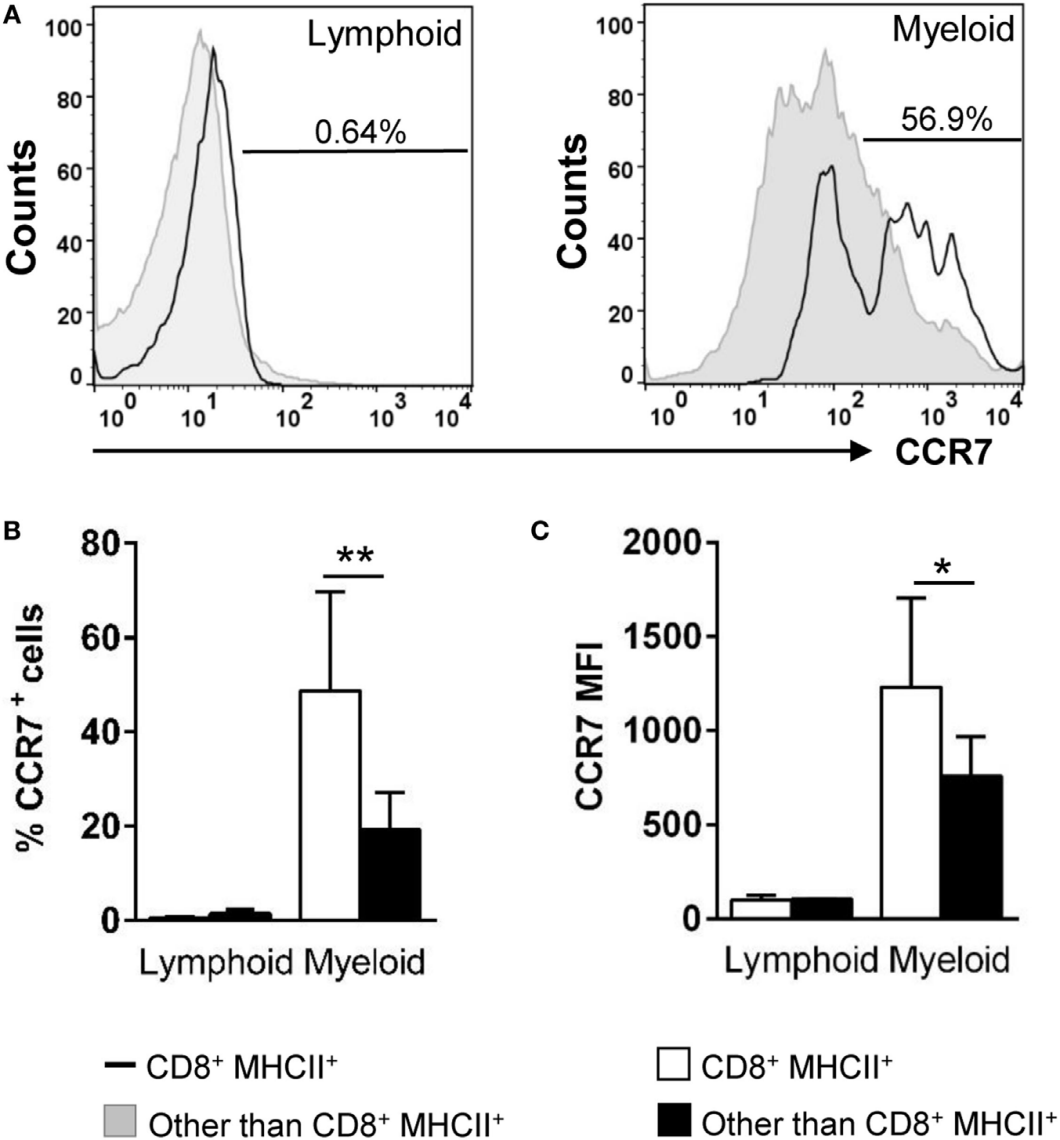
Expression of surface CCR7 on gill CD8^+^ dendritic cells (DCs). Trout leukocytes from gills were isolated, stained with specific Abs against CD8α, major histocompatibility complex (MHC) II and CCR7, and analyzed by multicolor flow cytometry. **(A)** Cells were gated as lymphoid and myeloid on the basis of their FSC and SSC. Then, CD8^+^MHC II^+^ cells were further gated on those populations, and CCR7 fluorescence was determined in CD8^+^ DCs (open line histograms) and compared against the general CCR7 levels in each gate (filled line histograms). Numbers on plots correspond to the percentage of CCR7^+^ cells after exclusion of negative cells present on the isotype controls. Average percentage of CCR7 cells **(B)** and MFI of CCR7 expression **(C)** in gill lymphoid and myeloid populations were calculated. Data are representative of nine individual fish from three independent experiments (*p ≤ 0.05 and **p ≤ 0.01).

In the mammalian respiratory system, the mobilization of DCs from the lungs to draining LNs is dependent on the chemokine receptor CCR7 and its ligands (34). Because there are no LNs in teleost fish, it has been proposed that secondary immune responses are locally orchestrated in the mucosal tissues that host APCs, B and T cells dispersed throughout the tissue (35). In this context, we decided to evaluate the levels of CCR7 surface expression in gill CD8^+^ DCs. In trout gills, no CCR7^+^ cells were found within the lymphocyte compartment (Figures 4A,B). On the other hand, the percentage CCR7^+^CD8^+^ DCs was significantly high (around 50%) compared with the amount of CCR7^+^ cells in the rest of the myeloid compartment (around 20%) (Figures 4A,B). In addition, a significantly higher CCR7 mean fluorescence intensity within the CD8^+^ DC subpopulation indicates that the density of CCR7 molecules on the cell surface is highest in this gill subset (Figures 4A,C).

### Gill CD8^+^ DCs Express TLRs and Cross-Presenting DC Markers

**Figure 5:**
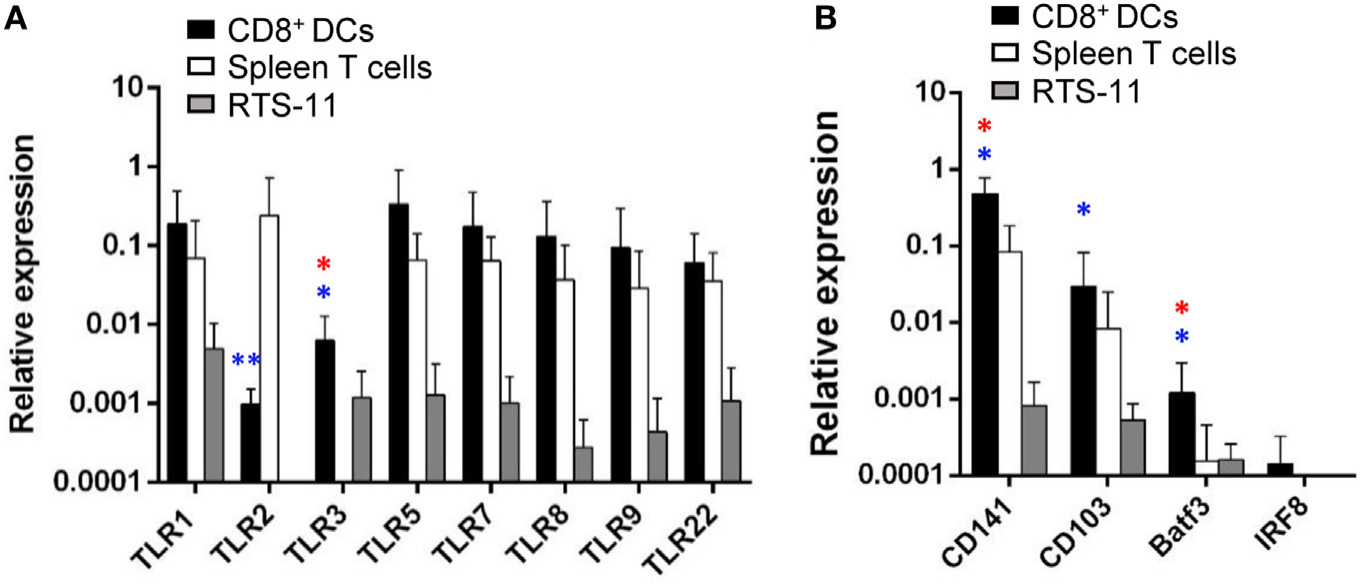
Transcription of toll-like receptor (TLR) genes and markers of mammalian cross-presenting dendritic cells (DCs) on gill CD8^+^ DCs. CD8^+^ DCs and splenic CD8^+^ T cells were isolated by flow cytometry, and RNA obtained. RNA was also obtained from the RTS11 monocyte–macrophage cell line. These RNAs were used to study the levels of transcription of the different TLRs **(A)** and other markers of cross-presenting DCs **(B)** by real-time PCR. Relative expression levels of the indicated genes to the endogenous control elongation factor 1α were calculated for each sample value (mean + SD; *n* = 3–7). Asterisks in red indicate significant differences between values obtained in gill CD8^+^ DCs and splenic CD8^+^ T cells, whereas asterisks in blue denote significant differences between values obtained in gill CD8^+^ DCs and RTS11 cells (*p ≤ 0.05 and **p ≤ 0.01).

Toll-like receptors are abundantly expressed on DCs since the recognition of molecular signatures from potential pathogens *via* TLRs is an essential step for DC activation that leads to the initiation of adaptive immunity (36). Hence, we analyzed the levels of transcription of all TLR genes identified in rainbow trout in gill CD8^+^ DCs, namely, TLR1, TLR2, TLR3, TLR5, TLR7, TLR8a, TLR9, and TLR22. TLR22 is a fish-specific cell surface sensor of dsRNA (37). Our results revealed that gill CD8^+^ DCs expressed high levels of TLR1, TLR5, TLR7, TLR8a, TLR9, and TLR22, together with intermediate levels of TLR2 and TLR3 (Figure 5A). The levels of expression of all TLR genes were much higher in gill CD8^+^ DCs than in RTS11 macrophages (Figure 5A). Concerning splenic CD8^+^ T cells, to our surprise, these cells also expressed high levels of most TLRs, with the exception of TLR3 (Figure 5A). As TLR3 is considered a signature marker for cross-presenting DCs including murine tissue CD103^+^ and lymphoid CD8^+^ DCs, and the human CD141^+^ DC lineage (16, 20, 21), our results point to the cross-presenting nature of our DC population. To further confirm this point, we examined the levels of transcription of additional exclusive markers associated with human and murine subsets of cross-presenting DCs. We confirmed that gill CD8^+^ DCs also transcribed CD103, CD141, Baft3, and IRF8 (Figure 5B), strongly suggesting that the CD8^+^ DC population identified in trout gills also corresponds to a cross-presenting DC subset.

### Gill CD8^+^ DCs Have IgM-Binding Capacity

**Figure 6:**
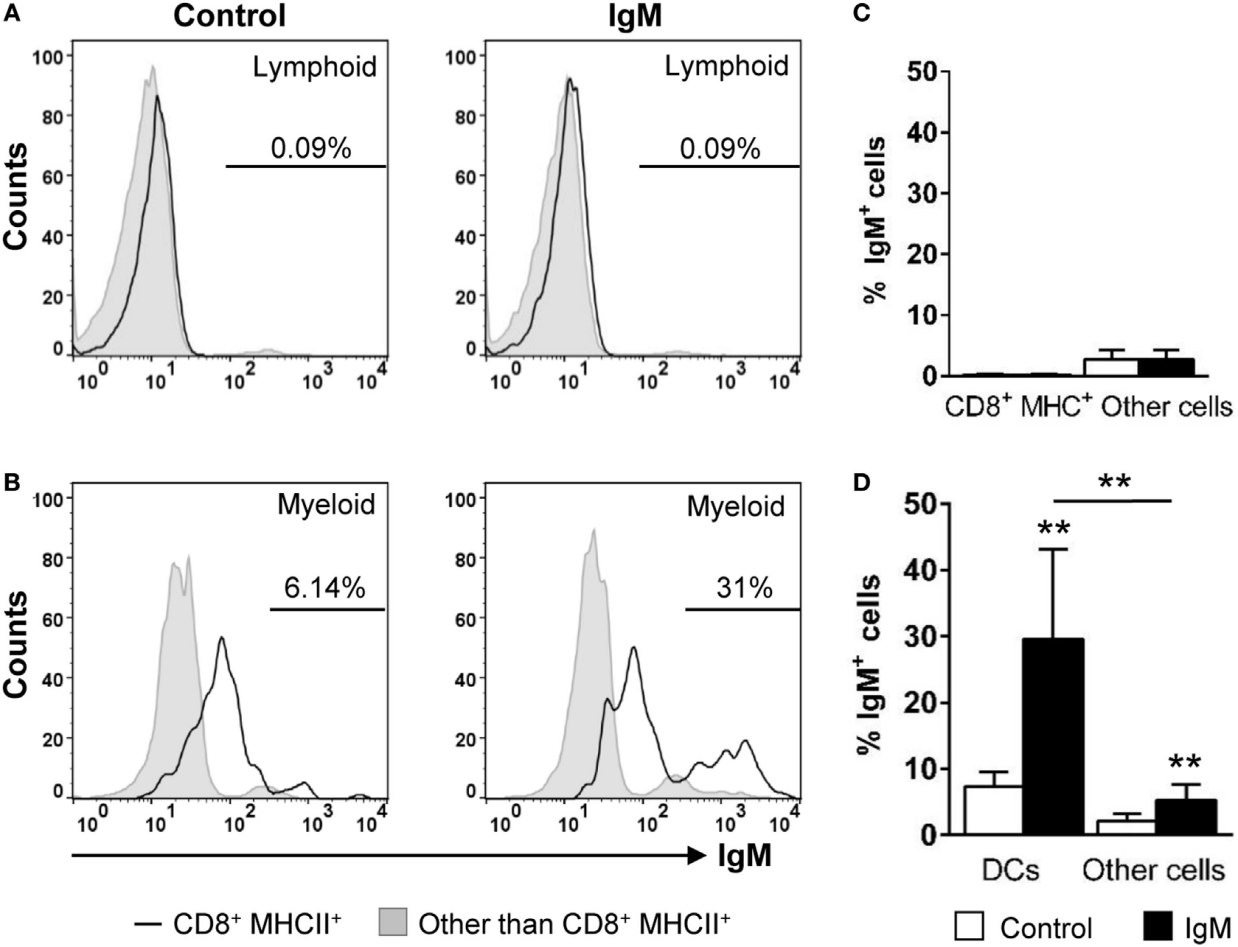
IgM-binding capacity of gill CD8^+^ dendritic cells (DCs). Gill leukocytes were incubated with purified IgM for 1 h at 20°C. Thereafter, cells were stained with specific Abs against CD8α, major histocompatibility complex (MHC) II, and IgM and analyzed by flow cytometry. Leukocytes were gated as lymphoid **(A)** and myeloid **(B)** cells on the basis of their FSC and SSC. Then, CD8^+^MHC II^+^ cells were further gated on those populations, and the IgM-binding capacity determined in CD8^+^ DCs (open line histograms) and compared against the general levels of IgM binding in lymphocytes and myeloid cells (filled line histograms). Data represent the IgM^+^ cells population after exclusion of negative cells present on the isotype controls. **(C,D)** Average percentage of IgM^+^ cells (left panels) and the MFI of IgM (right panels) within lymphoid **(C)** and myeloid **(D)** cells are shown. Data are representative of seven individual fish from two independent experiments (**p ≤ 0.01).

In mammals, it is known that most innate immune cells express Fc receptors that bind monomeric or aggregated Igs, immune complexes, and opsonized (antibody-coated) particles or cells (38). In DCs, engagement of FcR induces maturation and dramatically increases the efficiency of cross-presentation (39). As IgM is the prevalent Ig in the trout serum and gill mucus (40), we decided to analyze the capacity of the gill CD8^+^ DC subset to bind serum IgM. Our data revealed that ~31% of CD8^+^ DCs were able to bind purified IgM, while only ~5% of the rest of the cells within the myeloid gate bound IgM (Figures 6B,D). In the case of the cells contained within the lymphoid gate, there were a number of positive cells for IgM staining that should correspond to IgM^+^ B cells present in the gills (~2%); however, the IgM-binding capacity was negligible in lymphoid CD8^+^MHC^+^ cells from this gate (Figures 6A,C).

### Gill CD8^+^ DCs Produce and Are Activated by CD40L

**Figure 7:**
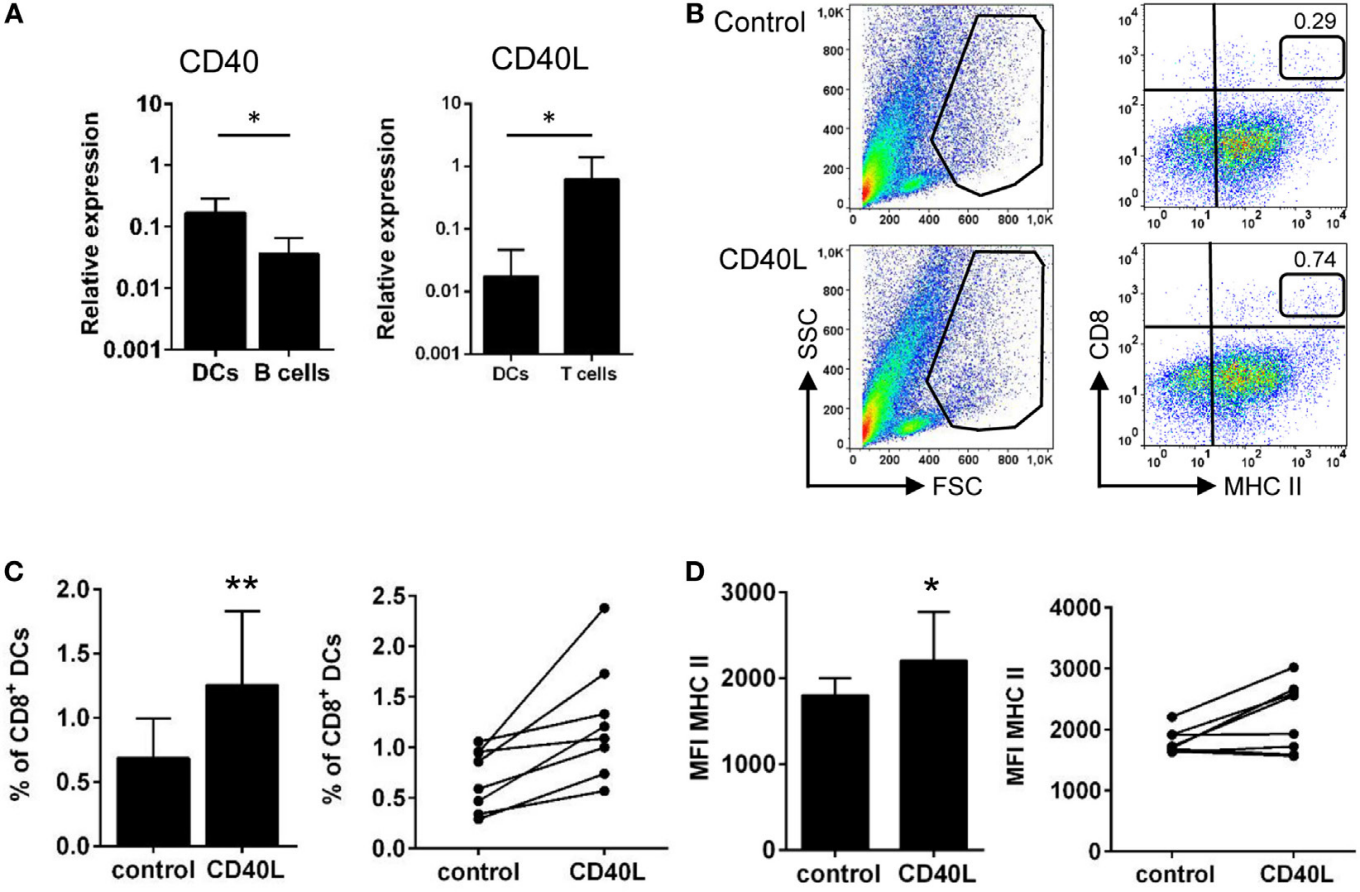
CD40 ligand (CD40L) stimulates gill CD8^+^ dendritic cells (DCs). **(A)** CD8^+^ DCs from gills, together with T cells and IgM^+^ B cells from spleen, were isolated by FACS sorting, and total RNA was obtained. The relative expression of CD40 and CD40L to the endogenous control elongation factor 1α was calculated for each sample, and mean (+SD) values from two experiments, involving three animals per experiment, were calculated. Asterisks denote statistically significant differences between indicated groups. **(B)** Gill leukocytes were incubated with CD40L during 48 h and then stained with anti-CD8α and anti-major histocompatibility complex (MHC) II mAbs. FSC/SSC profiles are shown (left), and a gate for myeloid cells was defined. Two-color CD8/MHC class II dot plots of myeloid gated cells are also shown (right panels). Percentage of myeloid CD8^+^MHC II^+^ cells among the total number of cells in each gate is shown in the upper right corner. Average percentage of CD8^+^ DCs in control medium or treated with CD40L was calculated **(C)**, as well as the MFI of MHC II on these populations **(D)**. The percentage of CD8^+^ DCs and the MFI of MHC II are also shown for each individual fish. Data are represented as mean + SD (*n* = 6). Asterisks denote statistically significant differences between the values obtained in the CD40L-treated group and the corresponding untreated group (*p ≤ 0.05 and **p ≤ 0.01).

It has been previously reported that engagement of CD40 by CD40L makes DCs more effective APCs, inducing the upregulation of MHC II and co-stimulatory molecules such as CD80 and CD86 (41). Thus, we aimed to analyze whether fish DCs respond to CD40L stimulation. As a first step, we analyzed whether DCs transcribed CD40 as well as CD40L, comparing their levels of expression to those observed in FACS isolated splenic IgM^+^ B cells or splenic T cells, respectively. We found that gill CD8^+^ DCs expressed CD40 transcripts at similar levels than those observed in isolated IgM^+^ B cells (Figure 7A), suggesting their responsiveness to CD40L. Surprisingly, gill CD8^+^ DCs also transcribed CD40L but the levels of transcription were 100-fold less than those seen in splenic T cells (Figure 7A). After the incubation of gill leukocytes with CD40L, the percentage of CD8^+^ DCs increased (Figures 7B,C). Considering that the mortality of total leukocyte populations from gills was similar in control and CD40L-treated cells (data not shown), our results indicate that CD40L increased the survival of CD8^+^ DCs. In addition, when gill leukocytes were treated with CD40L, the levels of expression of surface MHC II significantly increased on the CD8^+^ DC population (Figures 7B,D). To establish that the effects of CD40L were specific, we tested in parallel an irrelevant protein with a similar molecular weight and an N-terminal His tag obtained in the same conditions (C-His). This protein produced no effect on the survival or MHC II expression of CD8^+^ DCs (Figure S4 in Supplementary Material).

## Discussion

Dendritic cells are the most efficient APCs at initiating antigen-specific immune responses, inducing the differentiation of both naive CD4^+^ and CD8^+^ T cells (42). Among them, some specific DC subsets have the capacity to cross-present antigens, that is, to process exogenous antigens to enter the MHC I pathway, an essential process for the generation of optimal CD8^+^ T cell responses against intracellular pathogens and tumors (11). In mammals, DC subsets differ in morphology, anatomical location, surface markers, use of transcription factors and functionality, and in consequence, this divergence has greatly complicated the identification of homologous DC populations in different species. Despite this, it seems that the different DC subsets which possess cross-presenting capacities in mice and humans share some common signature markers including XCR1, TLR3, CLEC9A, Baft3, IRF8, and CADM1 (16) that suggest a common ancestor for all cross-presenting DCs despite the divergence in the expression of surface markers in different populations in mice and humans. Consequently, the identification of a CD8^+^ DC in rainbow trout skin expressing a range of these markers, namely, CD8, CD103, CD141, TLR3, Baft3, and IRF8, not usually expressed in other mammalian DC subsets (16, 20, 21), supports the hypothesis of these cells being a common ancestor for vertebrate cross-presenting DCs. In this study, we have demonstrated that such cells are also found in teleost gills, similarly to murine migratory CD103^+^ DCs (14) and human CD141^hi^ DCs (16) found in mammalian lungs.

We have established that this CD8^+^MHC II^hi^ cell type, which constitutes around 1–2% of the leukocyte population within what we have cataloged as a myeloid gate (cells with large size and high granularity), expresses DC markers such as DC-SIGN, CD83, and CD80/86 and negligible levels of expression of T-specific markers such as CD3 and TCRα. In our cytometric analysis, some cells with CD8 on the cell membrane and lower levels of MHC II surface expression are also found in the myeloid gate, but to parallel our previous studies in rainbow trout skin where this population was not so evident (10), we focused on studying the functionality of this CD8^+^MHC II^hi^ cell type, that we have designated as CD8^+^ DCs. However, future studies from our lab will be aimed at clarifying the nature of this CD8^+^MHC II^lo^ population, as it also seemed to respond to CD40L. In addition, it is worth noting that while the CD8^+^ lymphoid population in the spleen does not express MHC II, as expected for T cells, a high percentage of lymphoid CD8^+^ cells in gills expressed low levels of MHC II on the cell surface. Furthermore, these cells showed higher CD3 transcription levels than CD8^+^ DCs but also expressed some DC markers such as CD83 suggesting that it could correspond to a mixed population (data not shown). Interestingly, it has been shown that, in many species with the exception of mice, activated T cells express MHC II at their cell surface (43). Therefore, it could be plausible that T cells in a quite exposed mucosal tissue, such as the gills, are in a more activated state than T cells from central lymphoid organs, and consequently express low levels of MHC II on the surface. Again, the nature of this lymphoid CD8^+^ cell population is something that warrants further investigation.

Supporting the phenotypic characterization of trout gill CD8^+^ DCs, these cells were also found to exhibit functional features of mammalian DCs such as a strong phagocytic capacity and the ability to activate T cell proliferation, being this characteristic property of DCs (42). As only mature human DCs show T cell-activating properties in an auto-MLR (44), our results suggest a certain degree of maturation of trout CD8^+^ DCs in the gills already in homeostatic conditions. This hypothesis correlates with our transcriptional data that revealed high levels of transcription of CCR7, BAFF, and CD40 along with intermediate levels of CD80/86 and CD83, being the latter only expressed upon DC maturation in mammals (45). In addition, the fact that gill CD8^+^ DCs expressed high levels of surface CCR7 was further confirmed in flow cytometry using a specific antibody. In fact, it is well known that DCs reprogram their expression of chemokine receptors during maturation, being CCR7 one of the chemokine receptors expressed on mature DCs that is absent in immature DCs (46). This profile, sometimes cataloged as “semi-mature” and believed to be responsible for the orchestration of tolerance (47), was also found in trout skin CD8^+^ DCs (10). Thus, our new evidences obtained in a different mucosal tissue suggest that in teleost fish, innate and adaptive immune responses are initially coordinated directly within the mucosal tissue. In this sense, CCR7, which in mammals guides the encounter of DCs with lymphocytes on the LN (48), may be one of the receptors implicated in retaining semi-mature DCs within these mucosal tissues that comprise a considerable local B and T population, thus allowing a local coordination of tolerance versus immune response.

Despite skin and gill DCs sharing many phenotypical and functional features, some differences were also evident. For example, skin CD8^+^ DCs showed undetectable levels of transcription of CD8β (10), whereas CD8β mRNA levels were high in gill CD8^+^ DCs, in line with previous studies that showed the presence of CD8α^+β+^ DCs in the respiratory tract of mice (49). On the other hand, the mRNA levels of LAMP3, a tetraspanin known to play an important role in the phagocytic capacities of DCs (50), were very high in skin CD8^+^ DCs (10) but were nearly undetectable in the equivalent population in gills. Nevertheless, despite this difference in LAMP3 levels, the phagocytic capacity of both DC populations was similar, suggesting that the different LAMP3 transcription levels could have an effect on other immune functions modulated by this molecule such as cell migration (50). On the other hand, it could be possible that gill CD8^+^ DCs have slightly different mechanisms to undertake phagocytosis. Finally, the levels of CCR7 surface expression were much higher in gill CD8^+^ DCs (MFI ≈ 1,200) in comparison to those of the skin CD8^+^ DC population (MFI ≈ 300). All these evidences suggest an adaptation of teleost DCs to different mucosal tissues or the fact that the DCs are in different maturation status depending on their location.

After confirming the nature of this CD8^+^ DCs, we decided to study whether, as occurred in skin CD8^+^ DCs, these cells expressed specific markers of cross-presenting DCs. Gill CD8^+^ DCs transcribed all TLRs studied, including TLR3, a signature marker for cross-presenting DCs (16, 20, 21). Interestingly, multiple studies suggest that signaling through intracellular TLRs (TLR3, 7, and 9) improves antigen cross-presentation (51, 52). In addition, our results show that trout gill CD8^+^ DCs transcribed high levels of the surface markers CD141 and CD103, as well as Batf3 and IRF8, two transcription factors required for the development of mouse cross-presenting CD8^+^ DCs (53). Thus, all together, our results strongly point to this DC population as a common ancestor of cross-presenting DCs found in mammalian respiratory surfaces.

In this context, we decided to explore additional characteristics of this DC population, not previously investigated in teleost DCs. We have verified that gills CD8^+^ DCs have the capacity to bind IgM, most probably through the expression of surface Fc receptors that allow them to sense surrounding Igs as well as to sense and acquire opsonized antigens. In mammals, it has been shown that IgG immune-complexed antigens enter the cross-presentation pathway on mammalian DCs through the FcγR (54). Thus, the presence of Fc receptors on trout CD8^+^ DCs seems to correlate with the hypothesis of these cells being able to carry out antigen cross-presentation. Finally, we have also established that gill CD8^+^ DCs express CD40 and that the activation of this receptor through CD40L stimulation increases their survival and their levels of expression of MHC II. These results are in line with previous results observed in mammals where CD40L has been shown to have positive effects on DC activity through different mechanisms that include increasing the levels of expression of MHC II or co-stimulatory molecules [reviewed in Ref. (55)]. Strong effects of CD40 ligation on DC activity have also been reported for lung DCs in mice (56), probably as a result of high levels of antigen exposure in the respiratory mucosa, given the fact that CD40 expression has been commonly used as a marker to distinguish between inactivated and activated DCs in mice, as its expression is upregulated on DCs after encounter with different antigens (55). Thus, in our experiments, once again, the high levels of CD40 transcription and the responsiveness to CD40L suggest that CD8^+^ DCs in teleost gills have already been activated by waterborne antigens.

In conclusion, we have identified for the first time in teleost a specific subset of DCs in gills. These large CD8^+^MHC II^hi^ cells transcribed high levels of DC markers; had strong phagocytic and T cell-activating capacities; had the capacity to bind serum IgM; and were responsive to CD40L stimulation. Similarly to the equivalent population previously identified in rainbow trout skin, these CD8^+^ DCs found in rainbow trout gills expressed a wide range of molecules exclusive of different mammalian cross-presenting DC subsets. In consequence, these results indicate that the presence of cross-presenting DCs in respiratory surfaces preceded the appearance of tetrapods, highlighting the importance of these specific DC subsets throughout evolution.

## Ethics Statement

All of the experiments described comply with the Guidelines of the European Union Council (2010/63/EU) for the use of laboratory animals and have been approved by the Instituto Nacional de Investigación Agraria y Alimentaria (INIA) Ethics Committee (ORCEEA 2016-021).

## Author Contributions

IS performed all the experimental work, with help from AG and UF. CT and AG designed the experiments and wrote the main body of the paper, with contributions from IS and UF.

## Conflict of Interest Statement

The authors declare that the research was conducted in the absence of any commercial or financial relationships that could be construed as a potential conflict of interest.
