# Supplementary material for: Identification of a Potential Common Ancestor for Mammalian Cross-Presenting Dendritic Cells in Teleost Respiratory Surfaces
Source: Front Immunol. 2018 Jan 25;9:59. doi: 10.3389/fimmu.2018.00059 (PMC5788898; doi:10.3389/fimmu.2018.00059)
Supplement: Supplementary file 1 [file Data_Sheet_1.pdf]

Figure S1

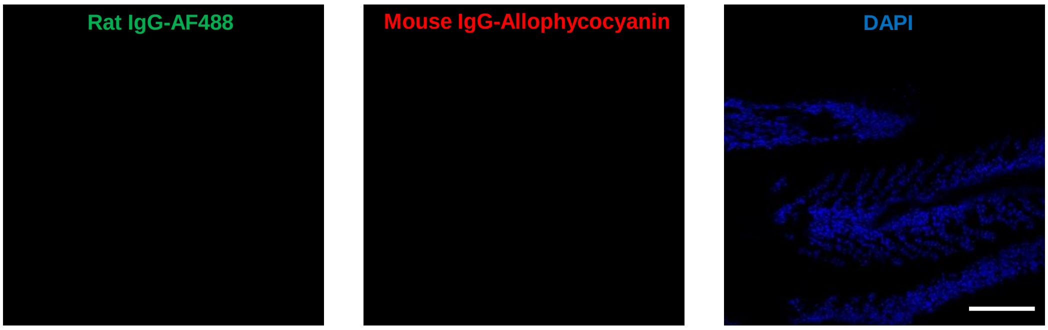

Figure S1. Isotype staining controls for Fig. 1B. Cryostat sections were prepared from rainbow trout gill, fixed, labelled with Isotype control antibodies AF488-labeled rat IgG and Allophycocyanin-labeled mouse IgG1, then counterstained with DAPI (blue) and analyzed by laser scanning confocal microscopy. A representative image is shown (bar=100 μm).

Figure S2

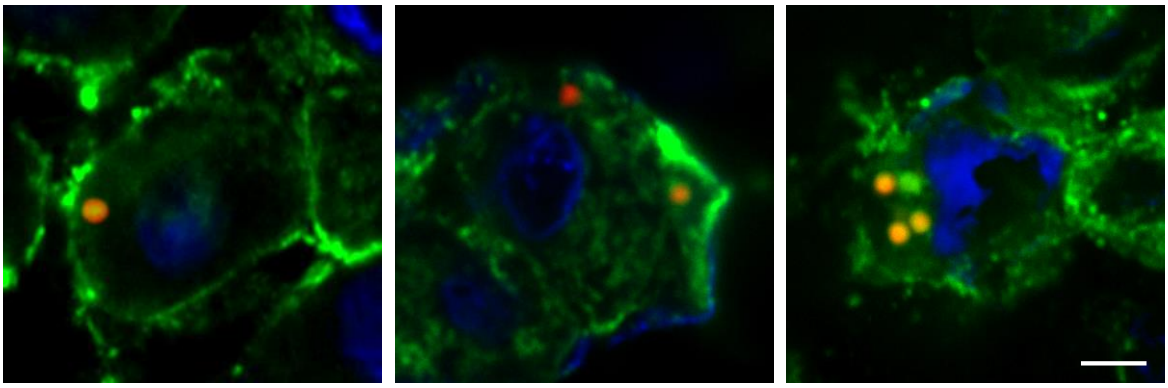

Figure S2. Confocal microscopy image of gill leukocytes phagocytizing labelled polystyrene beads. Leukocytes from gills were incubated with 1 µm diameter Crimson Red fluorescent polystyrene beads (shown in red) at a ratio of 1:10 (cell/beads) for 16 h. Non-ingested beads were removed in a glucose gradient and the cells were stained with a FITC-labeled anti-MHC II (green) counterstained with DAPI (blue) and analyzed by confocal microscopy. Note that all beads are found inside the cells. Representative images from 3 different fish are shown (bar=2 µm).

Figure S3

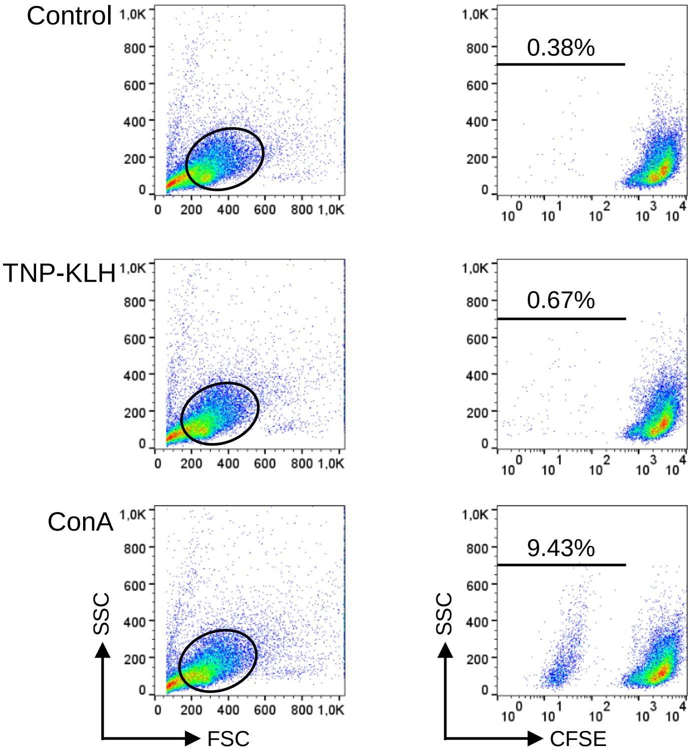

Figure S3. Splenic T cell proliferation rates in the absence of DCs. T cell-enriched cultured from splenocytes were FACS isolated and labeled with CFSE, as described in Material and Methods. This T cell-enriched fraction was cultured in the presence of control medium, TNP-KLH (5 µg/ml) or ConA (4 µg/ml) for 5 days, and then analyzed by flow cytometry to measure the level of CFSE dilution. Cell viability was assessed by using 7-AAD as described in Material and Methods.

Figure S4

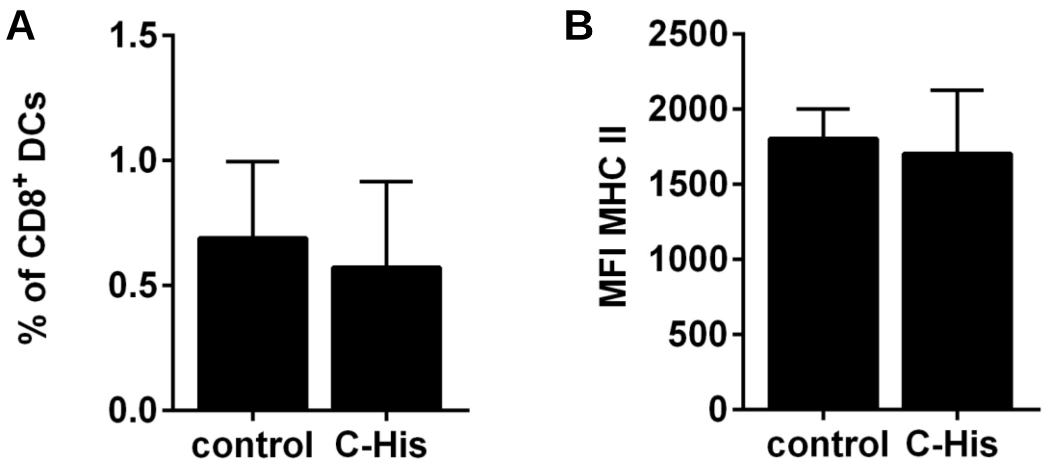

Figure S4. A control histidine tagged protein does not activate gill CD8^{+} DCs. Gill leukocytes were cultured in control medium or in the presence of an irrelevant histidine tagged protein (C-His, 5 μg/ml) during 48 h. Then, the cells were labeled with anti-CD8α and anti-MHC II mAbs. **(A)** The percentage of CD8^{+} DCs (defined as myeloid CD8^{+} MHC II^{+} cells) was determined as well as the MFI of MHC II on these populations **(B)**. Data are shown as mean + SD (n=6).

Table S1. Primers used in this study

Gene	Forward Primer	Reverse Primer	Accession no.
EF-1α	GATCCAGAAGGAGGTCACCA	TTACGTTCGACCTTCCATCC	AF498320
TCRα	ACGCACTTGGAATTATTCAACAAGA	GCTTCACATTTCTCTGAACCACCTA	OMU50991
CD3	CCTGATTGGAGTAGCTGTCTAC	GCTGTACTCAGATCTGTCCATGC	XM_021582112
CD8β	GTTCAAGGCCAGTAAAAGGGACAT	GCCTCCACAACTCGTTCTCTTTCT	NM_001124008.1
CD11b	GAAATTCACTGGGGATAGAGAAACAG	CTACTCCAACTCCTGTCCCTATTATC	AM713180.2
DC-SIGN	GAGAAGGAAGGGGATTGGAG	CCCATGTGATCCTCCTGACT	NM_001124633
CD83	GCTGTTGATAGCGGGAGGTA	TGTGGACTCAAGGCAATCTG	AY263793.1
CD80/86	GTGTTTCCTGGTTCTGGTATCTA	AACTTGCTGCTCCCTTTCCTC	FJ467621
BAFF	ATGTTTGATGCTTATTCTGGCAGG T	TGGGACTGTGTCTTGACTGTGTGTA	DQ218467
TLR1	CAGACGCCCTGTTGATGTT C	CCTTCACAAGTTCCACCACG	NM_001166101
TLR2	GATCCAGAGCAACACTCTCAACAT	CTCCAGACCATGAAGTTGACAAAC	XM_021578334
TLR3	AGCCCTTTGCTGCCTTACAGAG	GTCTTCAGGTCATTTTTGGACACG	NM_001124578
TLR5	TTGACTTATCTTCCAACGGATTCA	CTTTGAAATTGCTGAAACCAAATG	NM_001124208
TLR7	TACAGCTTGGTAACATGACTCTCC	CAACTCTCTGAGACTTGTCGGTAA	GQ422119
TLR8a	CATCTATGTTCTCATCCAGCAACC	GGTCCCCCTAATAGACAACCTCTT	GQ422120
TLR9	TCTTCATAGAGCTGAAGAGGCCTCA	GTTCCCACTGAGGAGAAGTGTTTT	NM_001129991
TLR22	TGGACAATGACGCTCTTTTACC	GAGCTGATGGTTGCAATGAGG	NM_001124412
CD141	CAGAATTCAGCAACTGGAAAGACAA	ACTTTTTCCTGACAAGGTCGTTCTG	KP203844
CD103	AGGAGTGATCTTAAAACACCCCAAG	TGGCAGACACAACACTGTAACCTAA	CDQ67442.1
Batf3	CACAGAGAGCAGATGAGTTGCATAA	TTGCTCCTCAGACAGAAACTGTACC	CA345618
IRF8	CCGAGGAGGAGCAGAAGAGTAAAAG	GCGGCATTGAAAGAACCCAT	AJ829674
CD40	TGGACTTGAATCTTAAGAGGGGAAC	GATGGCTCTCCAAATGGGATTATAG	GQ169787
CD40L	GAGTGTGAGAAAGACAGCCAGTCAG	CGTTTGACAGCTTTTCCTTCAACTT	EF160131
